# The effects of gut microbiota on appetite regulation and the underlying mechanisms

**DOI:** 10.1080/19490976.2024.2414796

**Published:** 2024-11-06

**Authors:** Miao Yu, Bing Yu, Daiwen Chen

**Affiliations:** a Animal Nutrition Institute, Sichuan Agricultural University, Chengdu, Sichuan Province, China; b DadHank(Chengdu)Biotech Corp, Chengdu, Sichuan Province, China

**Keywords:** Intestinal microbiota, appetite regulation, mechanis, human health, animal production

## Abstract

Appetite, a crucial aspect regulated by both the central nervous system and peripheral hormones, is influenced by the composition and dynamics of the intestinal microbiota, as evidenced by recent research. This review highlights the role of intestinal microbiota in appetite regulation, elucidating the involvement of various pathways. Notably, the metabolites generated by intestinal microorganisms, including short-chain fatty acids, bile acids, and amino acid derivatives, play a pivotal role in this intricate process. Furthermore, intestinal microorganisms contribute to appetite regulation by modulating nutritional perception, neural signal transmission, and hormone secretion within the digestive system. Consequently, manipulating and modulating the intestinal microbiota represent innovative strategies for ameliorating appetite-related disorders. This paper provides a comprehensive review of the effects of gut microbes and their metabolites on the central nervous system and host appetite. By exploring their potential regulatory pathways and mechanisms, this study aims to enhance our understanding of how gut microbes influence appetite regulation in the host.

## Introduction

The intestinal microbiota plays a pivotal role in regulating the host’s physiological processes. Recent advancements in intestinal microbiota research have provided a deeper understanding of its involvement in appetite regulation. Appetite, a vital factor in maintaining overall well-being, can contribute to the development of metabolic disorders and mental imbalances when dysregulated. Numerous studies have highlighted the intricate interplay between the intestinal microbiota and the host, elucidating their ability to modulate appetite regulation through diverse pathways.

The intestinal microbiota constructs a complex microecosystem with a close interaction with the host. They not only affect the secretion of appetite hormones through metabolites, but also directly interfere with the central nervous system through neural pathways, thereby regulating the host’s eating behavior.^1,2^ In particular, the intestinal microbiota can mediate the metabolism of food components through various pathways, producing key metabolites that affect appetite regulation. These metabolites may affect appetite by activating specific receptors, regulating hormone secretion, or directly influencing neuronal activity.^3,4^ For example, some bacteria that produce short-chain fatty acids can increase the host’s feeling of fullness, while some bacteria that produce endotoxins may lead to increased appetite.^5,6^ In addition, the composition and diversity of the intestinal microbiota are also closely related to the host’s appetite regulation. Some studies have found that specific intestinal microbial species or strains can affect the host’s appetite by producing anorexigenic or orexigenic metabolites.^7,8^

Although we have found some associations between intestinal microbiota and appetite regulation, the specific regulatory mechanisms remain to be further explored. This review aims to systematically summarize the role of intestinal microbiota in appetite regulation, explore its possible molecular mechanisms, and focus on its potential clinical applications in the treatment of appetite-related diseases. By deeply understanding how intestinal microbiota affects appetite regulation, we can provide a strong scientific basis for the development of new treatment strategies in the future, thereby addressing health problems caused by appetite dysregulation.

## Appetite regulatory system

The appetite regulation system is a complex system involving multiple organs, hormones, and neural networks. It is typically divided into the central regulatory system and the peripheral system. These two systems collaborate and influence each other, jointly maintaining an individual’s appetite and energy balance. The central regulatory system integrates information from the peripheral system to regulate the secretion of appetite-related hormones and neuronal activity, thereby influencing feeding behavior. On the other hand, the peripheral system senses changes in the body’s state and provides feedback signals to the central system to adjust appetite regulation strategies.

### Central regulatory system

Appetite is regulated by the central nervous system, where short-term signals are transmitted by gastrointestinal hormones to control eating, while long-term signals are associated with adipose tissue and environmental cues. The central nervous system, hormones, and the vagus nerve collectively constitute a complex appetite regulatory system that manages food intake. Imbalances in this system can lead to eating disorders and metabolic diseases.

The hypothalamus and the nucleus of the solitary tract (NTS) primarily regulate appetite and energy intake. The NTS, located within the hypothalamus, is a crucial nucleus that receives and integrates signals from peripheral organs, particularly the digestive tract, and the central nervous system to participate in appetite regulation. The NTS receives hormonal and neural signals from the digestive tract through connections to peripheral tissues such as the gastrointestinal tract and adipose tissue. These signals can include information about satiety and energy homeostasis. Additionally, the vagus nerve, a critical neural pathway connecting the digestive tract and the NTS, influences the NTS by carrying signals related to gastric distension, intestinal motility, and other aspects.

**Figure 1.**
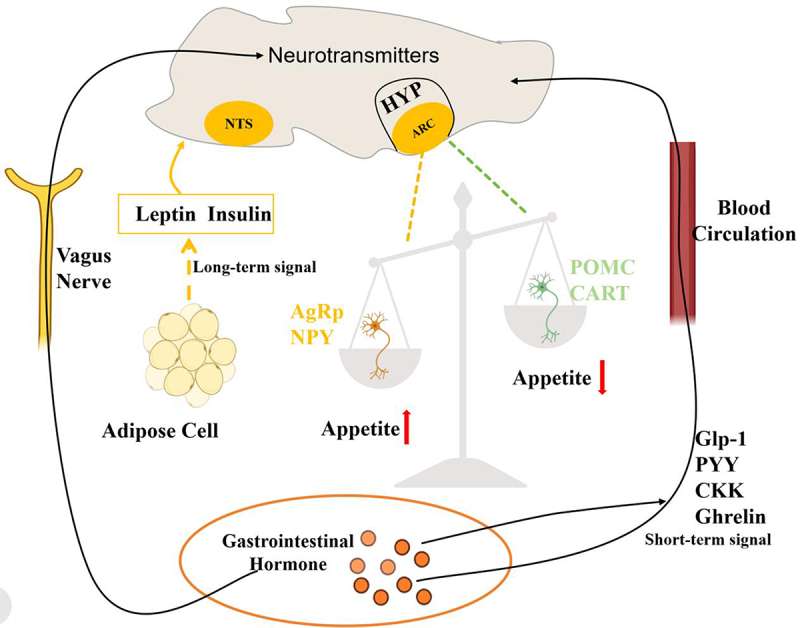
Appetite Regulation System HYP:hypothalamus; ARC:Arcuate Nucleus; NTS:Nucleus Tractus Solitarius; AgRp:Agouti-related Protein; NPY:Neuropeptide Y; POMC:Proopiomelanocortin; CART:Cocaine and amphetamine-regulated transcript Peptide.

Neurons within the NTS are responsible for integrating information from different signal sources to determine the current hunger or satiety state. Furthermore, the NTS interacts with other hypothalamic nuclei, particularly the arcuate nucleus, to further regulate appetite. Within the arcuate nucleus, two distinct types of neurons can be found: orexigenic (promoting appetite) and anorexigenic (suppressing appetite). Orexigenic neurons express appetite-stimulating signal substances such as Neuropeptide Y (NPY) and Agouti-Related Protein (AgRP), which increase appetite and make individuals more inclined to consume more food. Conversely, anorexigenic neurons express appetite-suppressing signal substances like Proopiomelanocortin (POMC) and Cocaine and Amphetamine-Regulated Transcript Peptide (CART). These signal substances contribute to reducing appetite and make individuals more inclined to control food intake. The interaction between these two types of neurons in the arcuate nucleus creates a balance, and changes in this balance regulate appetite (Figure 1).

### The peripheral system

The peripheral system comprises peripheral organs such as the gastrointestinal tract and adipose tissue, which send signals to the central nervous system to regulate appetite by sensing food intake and digestion status, as well as secreting hormones. For instance, gastrointestinal hormones (e.g., CCK, GLP-1, PYY) are released after eating, inhibiting appetite and promoting satiety to reduce food intake. Adipose tissue, on the other hand, participates in long-term appetite regulation by secreting hormones like leptin, which helps maintain stable body weight (Figure 1).

**Figure 2.**
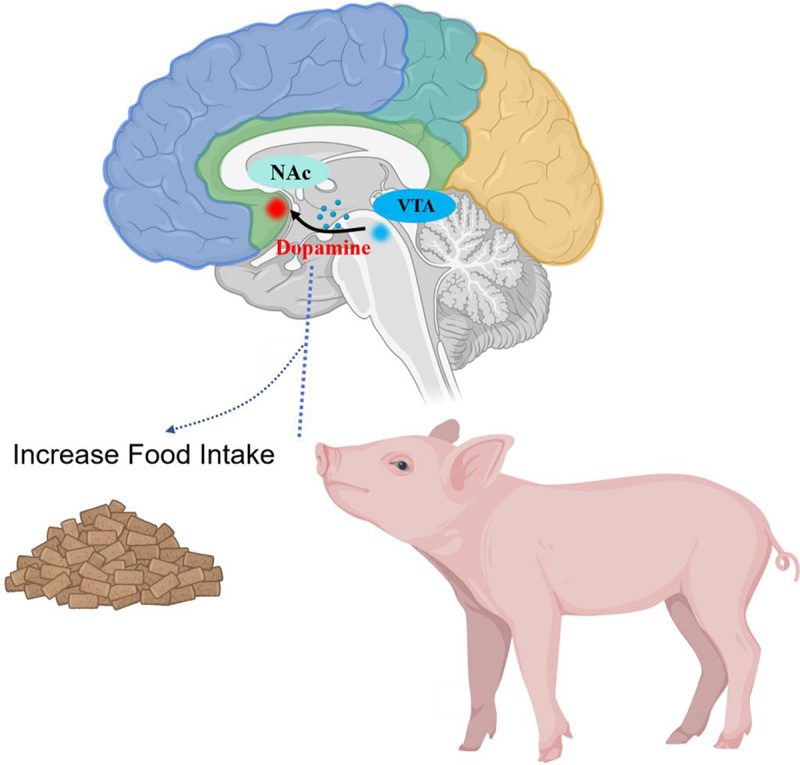
The reward mechanism of feeding NAc:Nucleus Accumbens; VTA:Ventral tegmental area.

Additionally, the dopaminergic reward system in the midbrain, which controls feeding reward regulation, can continuously promote appetite by providing a sense of satisfaction and pleasure. This system regulates appetite through the acquisition of reward and hedonic value (Figure 2).

## Intestinal microbiota and its main physiological functions

**Figure 3.**
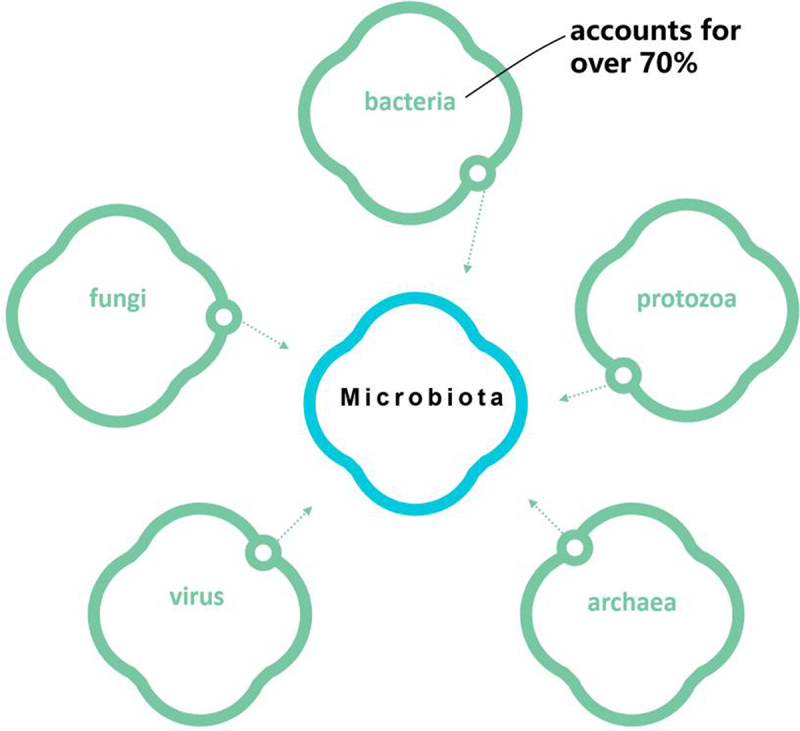
Gut microbial composition.

The intestines of humans and animals harbor a complex microbial community consisting of bacteria, fungi, viruses, archaea, and protozoa (Figure 3). Among these, bacteria dominate, accounting for over 70% of the microbial population, and together they constitute the intestinal microecosystem. The total number of intestinal bacteria in adults is approximately 3.8 × 10^13^, which is roughly equal to the number of their own somatic cells, collectively referred to as the intestinal microbiota.

The intestinal bacteria can be primarily categorized into several major groups: the Firmicutes, Bacteroidetes, Actinobacteria, Proteobacteria, and Verrucomicrobia phyla. Among these, the first two are the dominant microbiota in adult individuals, accounting for approximately 90% of all bacteria. It is generally believed that fetuses in the embryonic stage do not have intestinal microbiota. However, after birth and throughout the early life stages, bacterial colonization in the intestine occurs rapidly. During this period, the diversity of the intestinal microbiota is relatively low, and its composition undergoes significant changes, exhibiting a transition from aerobic to anaerobic bacteria. As the individual grows, the number and diversity of intestinal bacteria rapidly increase, and they gradually stabilize after the age of 2–3 years. Various complex factors such as disease, stress, environment, diet, and medication can all influence the composition and changes in intestinal bacteria.^9^

A mature intestinal microbiota gradually forms a secretory-immune regulatory network with the host organism, which is closely related to cognition, stress response, and social behavior. The intestinal microbiota participates in and mediates the information exchange of the “gut-brain” axis through multiple pathways, including the enteric nervous system, immune system, intestinal endocrine signals, and microbial metabolites. This significantly impacts the brain and behavior of the host.^10,11^ Research reveals that the presence of gut microbes affects intestinal stem cells and the mucosal immune system, which can cause intestinal disorders. Intestinal microbiota can regulate the rate of epithelial cell renewal of the host. Probiotics can help restore imbalance in an injured gut and promote its recovery.^12^

Studies have indicated that brain disorders such as depression and Parkinson’s disease are closely related to the imbalance of the intestinal microbiota.^13,14^ Increasing research confirms that the “microbiota-gut-brain” axis plays a crucial role in the regulation of appetite and eating behavior.

## Gut microbiota and the Central Nervous System

The gut microbiota maintains a tight communication with the central nervous system (CNS). These microorganisms not only participate in regulating nutrient absorption, metabolism, and immune responses, but also exert profound influences on the brain’s neural centers and host behavior. This communication between the gut and the brain is referred to as the gut-brain axis, a complex and sophisticated bidirectional information exchange system.^15^

Studies have revealed a significant interplay between the gut microbiota and the brain, exerting profound influences on behavioral patterns and brain functionality. Experimental results indicate that modifying the composition of gut microbiota in mice can regulate their behavioral and physiological states. By administering MDP (a metabolite produced by gut microorganisms) to mice, it has been demonstrated that MDP can alter their food intake behavior, activity levels, and neural activity patterns. This unveils the underlying mechanism of the interaction between gut microbiota and the brain, offering valuable insights for deeper investigations into the association between gut microbiota and the nervous system. Under various internal and external stimuli, the central nervous system affects the sensing, secretion, and motility functions of the gastrointestinal tract through efferent nerves. Conversely, the sensory state of the gastrointestinal tract can regulate the perception, emotion, and behavior of the central nervous system. In this process, gut microbiota play a crucial role. They facilitate the communication of information between the gut and the brain, and the signal molecules they produce may be transmitted to the central nervous system through various pathways, including the vagus nerve, peripheral circulatory system, and immune system, thereby influencing the host’s behavior.^16,17^

The vagus nerve, being the longest and most widely distributed pair of cranial nerves in the body, plays a crucial role in gut-brain communication. It is responsible for controlling functions of the respiratory, digestive, and other systems. Through afferent nerves, it is distributed in regions such as the duodenum and jejunum, receiving signals from gut microbiota. These signals can directly influence the function of the central nervous system, regulating the brain activity and behavioral manifestations of the host. For example, studies have shown that gut microbiota can regulate the anxiety behavior of the host by activating the vagus nerve. In mice supplemented with lactobacilli, their anxiety behavior was significantly reduced. When the vagus nerve is severed or loses its connection with the central nervous system, this anti-anxiety effect is no longer present, further confirming that signal peptides produced by gut microbiota may directly affect the function of the central nervous system through the vagus nerve.

The peripheral circulatory system, serving as a transport network within the organism, also plays a significant role in gut-brain communication. It delivers nutrients and oxygen absorbed and digested in the intestine to the brain, while also transporting key information molecules such as hormones, neurotransmitters, microbial metabolites, and immune signaling peptides to the central nervous system to regulate its function. Microorganisms have the ability to produce and recognize these neurochemicals, maintaining the stability of the internal environment of the host through interaction with it.^20,21^

The gut microbiota plays a pivotal role in the regulation of appetite, as they can modulate physiological processes including insulin sensitivity, glucose homeostasis, and the balance of energy intake through the production of metabolites like short-chain fatty acids (SCFAs). For example, adding fructose to the diet of Wistar rats can lower postprandial blood glucose concentrations and improve insulin sensitivity; while supplementing with inulin or β-glucan can reduce body weight and body fat in rats fed a high-fat diet and increase the number of Lactobacillus and Bifidobacterium species. One study found that GF mice consumed 29% more food than normal mice with microbiota, but their overall body fat content decreased by 42%.^18^

Colonizing GF mice with Bacteroidetes thetaioticus strains reduced food intake, increased liver fat synthesis from scratch, and body fat. Further research revealed that transplanting the cecal microbiota from healthy mice into GF mice caused significant elevations in blood leptin and insulin levels, and a two-fold increase in the concentration of 2-deoxyglucose 6-phosphate in the distal intestine. These combined results suggest that the absence of gut microbiota leads to reduced absorption of nutrients in the intestine, resulting in impaired anabolic metabolism, increased energy consumption, and elevated food intake in the host. Similarly, another experiment found that compared to normal mice with microbiota, GF mice had significantly elevated NPY and AgRP gene expression levels in the hypothalamus, along with significantly decreased POMC expression levels and serum leptin concentrations.^19^ Therefore, we have reason to believe that gut microbiota can control eating behavior by stimulating peripheral energy homeostasis signal changes and directly influencing the expression of central orexigenic neurons through the “microbiota-gut-brain” axis.

**Figure 4.**
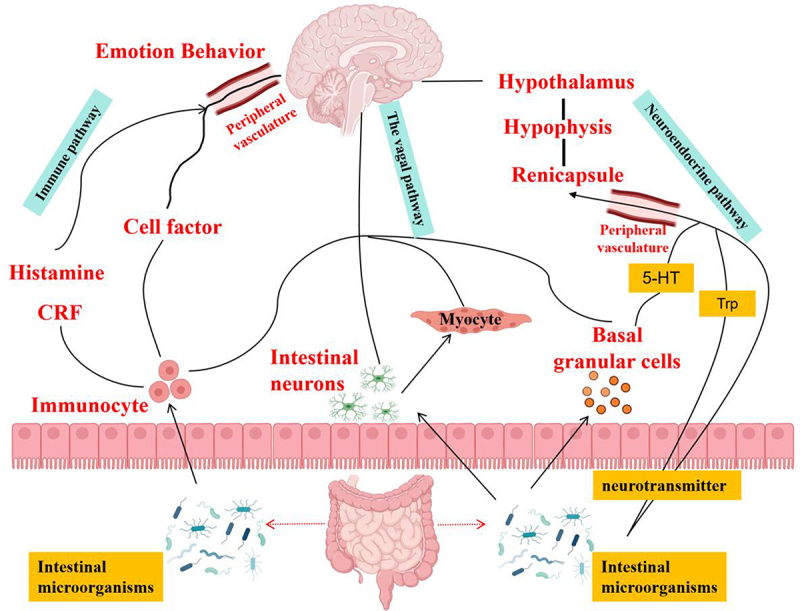
Information exchange between the gut and the brain.

These findings demonstrate that gut microbiota can participate in the regulation of host appetite through various pathways and maintain metabolic homeostasis. In summary, there is a complex interaction between gut microbiota and the central nervous system. They influence and regulate each other through the gut-brain axis, a bidirectional information exchange system, to jointly maintain the stability of the internal environment and the normal performance of behavioral phenotypes (Figure 4). As research in this field continues to deepen, we will gain a deeper understanding of the important role and mechanisms of gut microbiota in host health and disease development.

## Mechanisms by which gut microbiota influence appetite

### Gut microbiota influencing the secretion of appetite hormones

The physiological regulation of appetite is a complex process that involves the interaction of various circulating appetite-stimulating and appetite-suppressing hormones. These hormones are primarily produced by peripheral organs such as the gut, adipose tissue, and pancreas. Herein, we focus on the impact of changes in gut microbiota composition on appetite-related hormones, which play a crucial role in regulating brain behavior and function through humoral and neural pathways.

**Figure 5.**
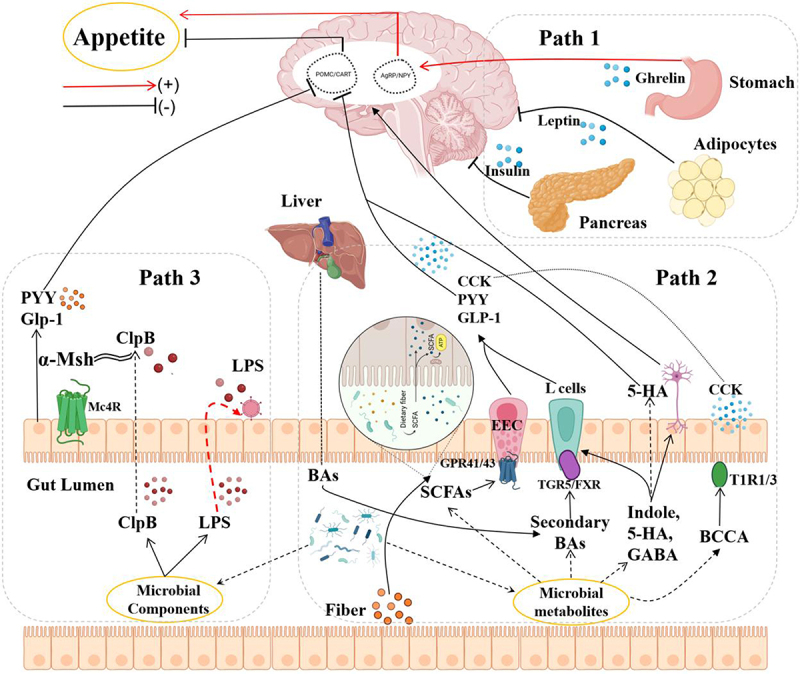
Mechanisms by which gut microbes influence appetite.

There is a substantial amount of hormones, peptides, and cytokines involved in appetite regulation that circulate in the human and animal bodies. Among them, one category is acute, short-term hunger and satiety signals secreted by the gastrointestinal tract before and after eating, including gut hormones such as Ghrelin, CCK, GLP-1, and PYY (Figure 5). These hormones play a key role in sensing and regulating energy intake. Another category is leptin and insulin, which are positively correlated with body fat tissue content. Leptin is produced by adipose tissue, while insulin is produced by the pancreas. Both are closely related to the long-term regulation of energy balance. These hormones regulate body weight and appetite by influencing metabolic processes and energy expenditure. These appetite factors transmit peripheral signals to the central nervous system (CNS) through humoral, immune, or neural pathways, activating and regulating appetite control signals in areas such as the hypothalamus and brainstem. The gut microbiota’s colonization, composition, and status can impact the autocrine, paracrine, and endocrine processes of peripheral hormonal factors, thereby contributing to the modulation of food intake.

#### Ghrelin

Ghrelin is an orexigenic peptide hormone secreted by X/A cells in the stomach and intestine. It plays a crucial role in transmitting hunger signals from the periphery to the central nervous system, stimulating food intake and regulating satiety (Figure 5). Ghrelin is composed of 28 amino acids, and the octanoylation of the serine residue at the third position is essential for its binding to the growth hormone secretagogue receptor and crossing the blood-brain barrier.^22^ Current research has identified two pathways for Ghrelin to stimulate appetite: firstly, peripheral Ghrelin can cross the blood-brain barrier and reach the hypothalamus, directly acting on NPY/AgRP neurons in the hypothalamus. Secondly, Ghrelin binds to receptors on the terminals of vagus afferent fibers, transmitting hunger signals to the brain and then to NPY/AgRP neurons in the hypothalamic neurons.^22,23^

Studies have indicated that gut microbiota can affect blood Ghrelin levels. There is a positive correlation between circulating Ghrelin and the number of Bacteroidetes and Prevotella species in the intestine, while there is a negative correlation with the number of Bifidobacterium, Lactobacillus, and Eubacterium rectale species.^24^ Another study found that transplanting fecal microbiota from healthy adult pigs to newborn piglets significantly promoted piglet growth and fat deposition, increased intestinal bacterial diversity and altered the composition of the microbiota, and increased the abundance of fiber-utilizing bacteria such as Prevotella.

Additionally, the blood Ghrelin levels of the recipient piglets increased significantly, GLP-1 levels also increased significantly, while CCK levels did not change significantly.^25^ Torres-Fuentes C. et al. recently reported that both short-chain fatty acids (SCFAs) and bacterial supernatants producing SCFAs inhibited Ghrelin signaling. They demonstrated that SCFAs disrupt Ghrelin-mediated calcium mobilization in vitro and inhibit Ghrelin signaling. This result suggests that circulating SCFAs may affect the Ghrelin pathway in nodose neurons that co-express FFAR3 and Ghrelin receptors.^26^

Furthermore, recent studies have shown that gut microbiota participate in the regulation of appetite by modulating Ghrelin-related signaling pathways.^27,28^ For instance, the administration of prebiotics, such as inulin and oligofructose, can suppress food intake by stimulating the production of glucagon-like peptide-1 (GLP-1) and peptide YY (PYY) while inhibiting Ghrelin secretion in both obese and healthy adults.^29,30^ In chicken blood monocyte experiments, the TLR9 agonist CpG-oligodeoxynucleotide significantly upregulates IL-1b in vitro.^31^ Additionally, CpG motifs have been detected in certain probiotics such as Spirulina.^32^ Taken together, these findings suggest that certain probiotics, such as Spirulina, may decrease Ghrelin levels by increasing CpG motif-stimulated IL-1b levels, leading to reduced food intake.

In summary, Ghrelin plays a crucial role in appetite regulation and its levels are influenced by gut microbiota, short-chain fatty acids, and probiotics. Modulating these factors can potentially affect Ghrelin signaling and thereby regulate appetite and food intake. Future studies are needed to further elucidate the mechanisms underlying these interactions and their potential applications in weight management and other related health conditions.

#### Leptin

Leptin is a 167-amino acid product produced by the obesity gene and is primarily secreted by adipose tissue as an anorexigenic hormone that transmits satiety signals to the central nervous system.^33^ Leptin reduces food intake by upregulating anorexigenic signals such as POMC and MC4R and downregulating orexigenic signals such as NPY and AgRP^34^ (Figure 5). Both overweight individuals and clinical animal models exhibit higher leptin levels compared to lean individuals, and plasma leptin levels are highly correlated with body mass index (BMI).^35^ Interestingly, despite elevated leptin levels in obese individuals, the signal for satiety is not transmitted due to leptin resistance.^36,37^ Therefore, improving leptin sensitivity and production can promote weight loss by reducing food intake.

Leptin is primarily secreted by white adipose tissue and reflects the body’s energy reserves.^38,39^ The stomach and intestine are also sources of leptin and contain leptin receptors.^40,41^ Leptin can cross the blood-brain barrier (BBB) and activate leptin receptors on two subpopulations of neurons in the arcuate nucleus (ARC) of the hypothalamus. Specifically, leptin activates neurons expressing pro-opiomelanocortin (POMC), which promote satiety, and inhibits neurons expressing neuropeptide Y (NPY) and Agouti-related protein (AgRP), which stimulate appetite, thereby inhibiting the host’s desire to eat.^42–46^ Zhang XL, et al. also found that leptin production was stimulated by the administration of soymilk fermented by B. bifidum, L. casei, and L. plantarum, but not soymilk alone. These results suggest that metabolites of these probiotics, such as short-chain fatty acids (SCFAs), could stimulate leptin production, leading to reduced food intake and body weight loss.^47^

Evidence from rodent experiments suggests a close association between the richness and diversity of gut microbiota and leptin signaling. For example, studies have found a significant correlation between lower bacterial diversity and higher circulating leptin concentrations in both obese and non-obese individuals^48^ Furthermore, both in vivo and in vitro experiments have revealed that gut microbiota can affect energy metabolism in obese individuals and mice by increasing intestinal permeability and translocation to adipose tissue, primarily through the inhibition of leptin signaling.^49,50^ Notably, leptin treatment in germ-free mice reduced the expression of NPY and AgRP in the hypothalamus, but had no significant effect on wild-type mice, further confirming the crucial role of gut microbiota in leptin signaling.^51^ More in-depth studies have shown that the removal of gut microbiota inhibits leptin signaling and feeding behavior in mice under normal chow conditions, but only suppresses feeding behavior without affecting leptin signaling under a high-fat diet.^52,53^ This suggests that the effect of gut microbiota on leptin signaling is modulated by the type of diet. Interestingly, the effects of probiotic or prebiotic supplementation on leptin signaling and feeding behavior are variable or even contradictory in genetically and diet-induced obese mice.^54–57^ These conflicting results may stem from the complex influence of functional changes in the microbial community on leptin signaling, and thus, the precise role of specific microorganisms remains to be further explored. Additionally, it is unclear whether probiotics or prebiotics would have similar effects in patients with eating disorders. To fully understand the relationship between gut microbiota and leptin signaling and its role in appetite regulation, future studies are needed to further investigate the changes in gut microbial communities under different dietary conditions and their specific mechanisms of action on leptin signaling.

#### CCK

CCK (cholecystokinin) is the first intestinal hormone identified to have a regulatory role in food intake.^58^ It is primarily released by duodenal enteroendocrine I cells in response to lipids and proteins in the diet, through G protein-coupled receptors GPR40 and calcium-sensing receptors. There are two subtypes of CCK receptors: CCK1 and CCK2, previously classified as CCK A and CCK B. Among them, the anorexic effect of CCK seems to be primarily mediated by CCK1 receptors on the vagus nerve.^59,60^ Both receptors are widely distributed in the brain, especially in the brainstem and hypothalamus regions.^61^

CCK exerts a direct influence on afferent neurons of the vagus nerve, primarily targeting the nucleus of the solitary tract and subsequently activating neural networks responsible for appetite control.^62^ Studies by Duca et al. have shown that CCK plays a crucial role in the transmission of satiety signals, particularly those mediated by lipids in the diet.^33^ They found that the lack of CCK-mediated satiety signals under a high-fat diet led to excessive calorie intake and weight gain.^33^ Therefore, increasing the sensitivity and concentration of CCK could be an effective strategy for the treatment of obesity.

The secretion of CCK by intestinal I cells can be stimulated through the activation of TLR4, TLR5, and Toll-like receptor TLR9. Bacterial LPS, flagellin, and CpG motifs can activate these receptors in intestinal I cells, respectively.^63^ These ligands are typically associated with pathogenic bacteria, so bacterial infections may trigger excessive production of CCK, leading to symptoms such as nausea and vomiting. However, interestingly, significant amounts of CpG motifs have also been detected in certain probiotic bacteria, such as Lactobacillus plantarum and Lactobacillus rhamnosus.^62^ This suggests that these probiotic strains may have the potential to stimulate CCK secretion and reduce food intake.

In addition to potentially stimulating CCK secretion, CpG motifs in probiotics may also improve mucosal function and the immune system, thereby promoting human health.^62^ In a zebrafish larva model, Lactobacillus rhamnosus upregulated the expression of CCK genes by modulating the gut microbiota composition and increasing the concentration of free fatty acids, stimulating the production of CCK.^64^ At the same time, the abundant CpG motifs in this probiotic may also promote the production of CCK. Other studies have shown that Lactobacillus acidophilus and Lactobacillus gasseri significantly enhanced CCK secretion by STC-1 cells in vitro.^65^

These research data suggest that certain lactic acid bacteria species may reduce food intake and achieve weight loss by stimulating the production of CCK. However, further studies are needed to validate their specific effects on appetite and weight in appropriate animal models.

#### PYY

Peptide YY (PYY) is a 36-amino acid peptide hormone initially isolated from porcine small intestine.^66^ This hormone is primarily released by L cells in the distal intestine and exists in two forms in circulation: PYY(1-36) and PYY(3-36). Among them, PYY(3-36) is the main circulating form, which is generated by the cleavage of the N-terminal tyrosine-proline residue of PYY(1-36) by the enzyme dipeptidyl peptidase IV (DPPIV). PYY(1-36) has affinity for all Y receptors, while PYY(3-36) primarily binds to hypothalamic Y2 receptors to exert its inhibitory effect on food intake.

Studies have shown that peripheral administration of PYY significantly reduces food intake and inhibits weight gain in rats. In humans, intravenous injection of PYY effectively suppresses appetite and food intake in both lean and obese individuals, indicating that the sensitivity to PYY is not altered in obese subjects. Notably, PYY levels are elevated in patients with gastrointestinal diseases such as inflammatory bowel disease and fatty liver disease.^67,68^

In addition to its effects on food intake, PYY also participates in regulating energy expenditure,^69,70^ delaying gastric emptying, and reducing gastric acid secretion. The anorexic action of PYY(3-36) may be achieved either by directly acting on the arcuate nucleus (ARC) of the hypothalamus or indirectly through signal transduction via the vagus nerve and brainstem. Studies have found that peripheral administration of PYY(3-36) increases the expression of c-fos in the ARC, which is a marker of neuronal activation, and direct injection of PYY(3-36) significantly suppresses food intake. This effect is likely mediated by Y2 receptors, as the anorexic effect of peripheral PYY(3-36) is blocked in mice lacking Y2 receptors.^71^ Despite conflicting results,^72^ studies have shown that bilateral subdiaphragmatic vagotomy and hypothalamic brainstem pathway ablation can eliminate the anorexic effect of peripheral PYY(3-36) on food intake in rats, emphasizing the importance of vagus nerve-brainstem signaling in the regulation of food intake by PYY.^73,74^

The anti-obesity effect and mechanism of Ligilactobacillus salivarius LCK11 (LCK11) is studied using a C57BL/6J male mouse model in which obesity is induced by a high-fat diet (HFD). Results show that LCK11 can prevent HFD-induced obesity, reflected as inhibited body weight gain, abdominal and liver fat accumulation and dyslipidemia. Analysis of its mechanism shows that on the one hand, LCK11 can inhibit food intake through significantly improving the transcriptional and translational levels of peptide YY (PYY) in the rectum, in addition to the eventual serum PYY level; this is attributed to the activation of the toll-like receptor 2/nuclear factor signaling pathway in enteroendocrine L cells by the peptidoglycan of LCK11. On the other hand, LCK11 supplementation effectively reduces the Firmicutes/Bacteroidetes ratio and shifts the overall structure of the HFD-disrupted gut microbiota toward that of mice fed on a low-fat diet; this also contributes to preventing obesity. LCK11 shows the potential to be used as a novel probiotic for preventing obesity by both promoting PYY secretion to inhibit food intake and regulating gut microbiota.^75^

Recent studies have also revealed a correlation between the gut microbiota and PYY levels. Qu L. et al. found that in a diabetic rat model, the concentrations of short-chain fatty acids (SCFAs) and PYY were lower than in the normal control group. Oral administration of lactic acid bacteria significantly increased the abundance of total SCFAs-producing strains, leading to increased SCFAs production and subsequently elevated PYY levels.^76^ Brooks L. et al. demonstrated that in normal, but not ffar2-deficient, mice, SCFAs increase PYY levels by upregulating Pax4, a critical transcription factor for l-cell differentiation. This suggests that activation of FFAR2 in l-cells by SCFA-induced signaling enhances PYY levels by stimulating l-cell differentiation.^77^ Additionally, SFCFAs-induced PYY production was significantly reduced in FFAR3 knockout mice, indicating that FFAR3 also plays an important role in PYY secretion.^78^ However, the underlying mechanisms remain to be further elucidated.

It is reported that the gut microbiota regulates plasma PYY levels by producing SCFAs and secondary bile acids. These secondary bile acids can bind to FFAR2/3 or G protein-coupled receptor TGR5 on l-cells.^77–79^ Notably, primary bile acids (BAs) are physiologically important steroid acids produced in the liver, and they play a crucial role in the absorption and transport of nutrients, especially lipids, in the intestine.^80^ In the small intestine, primary BAs are deconjugated by bile salt hydrolases produced by various intestinal bacteria. In the large intestine, unconjugated primary BAs are converted into unconjugated secondary BAs by intestinal bacteria, mainly including Clostridium scintillans and Lactobacillus.^81^ These microbial transformation processes may have profound effects on the secretion and activity of PYY, providing new insights for the treatment of obesity and related metabolic diseases.

In response to nutrient intake, GLP-1, along with the L-cells present in the intestine, simultaneously secretes PYY. GLP-1 receptors are expressed in both vagal afferent nerve endings and the hypothalamus, and their binding can induce a sense of satiety in the body.^82^ GLP-1 exists in two biologically active forms: GLP-1(7-37) and GLP-1 R(7-36) amide. Among these, GLP-1 R(7-36) amide is the primary circulating form found in humans.^82^ When GLP-1 binds to GLP-1 R, it stimulates adenylate cyclase activity, leading to the production of cAMP.^83^ GLP-1 R is widely distributed in the brain, gastrointestinal tract, and pancreas.^83,84^ The physiological effects of GLP-1 include reducing food intake, inhibiting glucagon secretion, and delaying gastric emptying.^85^ Intravenous infusion of GLP-1 can lead to a dose-dependent reduction in food intake in both normal-weight and obese subjects; however, obese subjects have a blunted postprandial GLP-1 response compared to lean subjects.^86^ In addition to its anorexic effects, GLP-1 has a potent incretin effect, stimulating insulin secretion in a glucose-dependent manner after carbohydrate intake.

SCFAs promote the secretion of GLP-1 from l-cells by activating FFAR2 and FFAR3, which is similar to the secretion mechanism of PYY.^78,87,88^ Patrice et al. also found that a non-digestible carbohydrate diet promotes l-cell differentiation in male Wistar rats and induces more endogenous GLP-1 secretion.^89^ Additionally, probiotics and mixed bacteria can alter the gut microbiota composition of HFD mice and promote GLP-1 secretion.^78^ Yadav H. et al. demonstrated that butyrate is a key factor in stimulating the release of GLP-1 from enteroendocrine cells.^78^ Zhao et al. conducted a randomized clinical trial showing that dietary fiber significantly increases the diversity and abundance of bacteria producing SCFAs. The altered gut microbiota results in increased production of acetate and butyrate, which subsequently stimulates the secretion of GLP-1 and PYY.^90^

LPS, as a major component of the outer membrane of Gram-negative bacteria, is also involved in regulating intestinal GLP-1 secretion. Lebrun et al. reported that after damage to the intestinal barrier, LPS from intestinal bacteria can easily leak into the bloodstream, leading to a rapid increase in plasma GLP-1 levels through a toll-like receptor 4 (TLR4)-dependent mechanism.^91^ Recent studies further suggest that LPS requires the expression of Gcg in the distal intestine to maximize plasma GLP-1 levels.^92^

In a study conducted on chickens,^93^ dietary supplementation with Clostridium butyricum, Bifidobacterium, Lactobacillus plantarum, Lactococcus faecalis, and a combination of guar gum probiotics altered cecal microbiota diversity, affected the content of short-chain fatty acids (SCFAs), and inhibited lipid production in the liver and abdominal adipose tissue. In intestinal epithelial cells (IECs), acetate, propionate, and butyrate enhances the expression of glucagon-like peptide-1 (GLP-1) by activating the MAPK pathway, particularly through the ERK and p38 MAPK pathways. GLP-1 then inhibited lipid accumulation in primary hepatocytes through the AMPK/ACC signaling pathway.

#### Insulin

Insulin is a protein hormone secreted by the beta cells of the pancreas that plays a crucial role in regulating carbohydrate and fat metabolism as well as maintaining blood glucose homeostasis.^94^ Beyond these classical physiological functions, insulin has also been found to act as a satiety signal involved in appetite regulation^95^ (Figure 5). Numerous studies have shown that insulin can stimulate the activity of POMC neurons while inhibiting the function of NPY neurons, thereby reducing food intake.^96,97^ Both human and mouse studies have demonstrated that the composition and diversity of the gut microbiota have a significant impact on insulin signaling. For example, individuals with lower gut bacterial diversity tend to exhibit higher insulin resistance,^98^ while insulin sensitivity is relatively higher in germ-free or gut microbiota-deficient mouse models.^99,100^

The gut microbiota interacts with the insulin signaling pathway through its metabolites, such as short-chain fatty acids (SCFAs), lipopolysaccharides (LPS), and branched-chain amino acids (BCAAs). Tang C et al. found that mice lacking FFAR2 and FFAR3 have increased insulin secretion, suggesting that SCFAs may inhibit insulin production by activating FFAR2/3.^101^ Interestingly, some studies suggest that certain SCFAs, particularly butyrate, may improve insulin sensitivity. In clinical studies, subjects receiving fecal microbiota transplantation from lean donors exhibited increased insulin sensitivity by increasing the abundance of butyrate-producing bacteria.^102^ Meanwhile, other data show that the use of vancomycin to reduce the abundance of butyrate-producing bacteria leads to a decrease in insulin sensitivity.^103^

Incorporating the above research, we can conclude that improving the population of butyrate-producing bacteria in the gut may potentially enhance insulin sensitivity and improve insulin resistance. Recently, Pedersen HK et al. also found that patients with insulin resistance had elevated serum levels of BCAA, with the key strains identified as Coprococcus eutactus and Bacteroides vulgatus. Their results showed that administering P. copri to high-fat diet (HFD) mice increased serum BCAA levels and led to insulin resistance.^104^ However, when P. copri was given to mice on a high-fiber and low-fat diet, it improved insulin sensitivity by producing succinate, a substrate for intestinal gluconeogenesis.^105^ These findings further emphasize the complex impact of specific gut microorganisms such as P. copri on insulin sensitivity under different dietary conditions.

In summary, hormones from peripheral organs play a crucial role in regulating appetite and related metabolic processes such as energy homeostasis and hedonic eating. In recent years, increasing research has pointed to the potential of gut microbiota alterations to regulate host feeding behavior by influencing the secretion of appetite-related hormones. However, this theory still requires rigorous clinical trials to validate its effectiveness and ensure that we have conclusive evidence to support it.

### Microbial metabolites

For a considerable period, it has been postulated that gut microbial metabolites play a pivotal role in energy production and the modulation of the microbiota-gut-brain axis, potentially influencing the physiological and psychological functions of mammals.^106,107^ A deeper comprehension of the interplay between gut microbial metabolites and appetite holds promise for the development of personalized nutritional strategies aimed at treating eating disorders. In the following, we summarize some additional microbial metabolites that are relevant to appetite control.

#### Short-chain fatty acids (SCFAs)

SCFAs, such as acetate, propionate, and butyrate, are primarily produced by gut bacteria through the fermentation of low-digestible polysaccharides, such as dietary fiber. Besides serving as a source of energy, SCFAs play crucial roles as signaling molecules, especially in appetite control.^108^ The addition of a 0.1% mixture of SCFAs to piglets’ feed significantly reduced their average daily feed intake, along with significant upregulation of serum concentrations of GLP-1, PYY, and leptin, as well as mRNA expression of FFAR2 in the colon and FFAR3 in the cecum.^107^ Similarly, Li et al.^109^ found that acute oral administration of 5% sodium butyrate to mice resulted in a sharp decrease in food intake, activation of adipose tissue, and decreased numbers of c-fos-positive neurons and NPY neuron activity in the ARC, NTS, and dorsal vagal complex.

In the large intestine, the gut microbiota produces SCFAs primarily including butyrate, acetate, and propionate through the fermentation of undigested dietary fiber^108^ (Figure 5). These SCFAs have been reported to have multiple benefits in controlling appetite and energy homeostasis.^110^ Roselli et al. found that oral administration of a probiotic mixture containing Lactobacillus acidophilus and B. bifidum, Lactobacillus casei, and Lactobacillus plantarum significantly increased the secretion of SCFAs and promoted the production of leptin.^111^ These findings indicate that the metabolites produced by these probiotics, including short-chain fatty acids (SCFAs), can stimulate leptin production, ultimately leading to decreased food intake and body weight reduction.^112^

SCFAs stimulate the production of plasma leptin by interacting with free fatty acid receptors (FFARs), namely FFAR2/GPR43 and FFAR3/GPR41^113^(Figure 5). Hong YH et al. demonstrated that SCFA-induced activation of FFAR2 in adipocytes stimulates adipocyte differentiation and increases leptin levels.^114^ Additionally, Xiong et al. were the first to show that activation of FFAR3 on adipocytes by SCFAs increases leptin production.^113^ However, recent studies using the same PCR primers failed to detect FFAR3 expression on adipocytes, suggesting that FFAR3 expression on adipocytes may be extremely low or undetectable.^114,115^ Nevertheless, in FFAR3 knockout mice, SCFA-induced leptin secretion is attenuated due to downregulation of FFAR2 expression.^115,116^ These results suggest a certain interplay between SCFA-induced FFAR3 activation and FFAR2. Therefore, the interaction between FFAR3 and FFAR2 in adipocytes remains to be further explored.

The relationship between short-chain fatty acids (SCFAs) and lactate-producing gut microbiota has been found to have both positive and negative effects, and is closely related to feed intake.^117^ In this study, 280 commercial Duroc pigs were reared, and their diurnal and nocturnal feeding behaviors during the 30-100 kg period were recorded.^117^ The findings revealed that pigs exhibiting a gut microbiota dominated by Prevotella exhibited a higher average daily feed intake (ADFI).^117^ SCFAs affect metabolism and appetite by binding to G protein-coupled receptors in various tissues and organs, such as FFAR3/GRP41 and FFAR2/GRP43.^70^ These receptors have opposing signaling effects.^118^ On one hand, SCFAs can activate insulin secretion and FFAR3 (FFAR3/GRP41), thereby activating signaling pathways related to ghrelin and inhibiting insulin secretion.^118–120^ However, their specific effect on appetite is not yet clear. On the other hand, the binding of SCFAs to FFAR2 (FFAR2/GRP43) can suppress appetite, further promoting the release of GLP-1, PYY, insulin, and leptin to signal to the appetite system.^28,121–123^ GLP-1 and PYY are important anorexigenic hormones^124–128^ that can cross the blood-brain barrier to activate POMC.^129^ In addition, GLP-1 and PYY can increase insulin sensitivity, slow gastric emptying and intestinal motility, thereby affecting appetite.^130–133^ Interestingly, acute colonic propionate delivery can reduce food intake and stimulate the secretion of PYY and GLP-1, while long-term colonic propionate delivery has less of an effect on PYY and GLP-1, which may be related to propionate tolerance.^73^ Clinical studies have shown that reward processing and pleasure responses are associated with the reduction in energy intake caused by deglycosylated mannan, rather than the secretion of PYY and GLP-1.^134^

After entering the blood circulation, gut-derived SCFAs can cross the blood-brain barrier to directly affect neurons related to appetite in the brain.^135^ For example, intraperitoneal injection of acetate can increase the expression of POMC in the hypothalamus, inhibit AgRP, and significantly reduce food intake without affecting the concentrations of circulating PYY and GLP-1. This suggests that acetate can directly generate a satiety signal.^136^ A study found that acetate produced by colonic microbiota fermentation of inulin in mice can cross the BBB into the CNS. After intraperitoneal injection of acetate, it can be rapidly absorbed by the brain, regulate the activity of orexigenic and anorexigenic neurons in the hypothalamus, reduce food intake, but does not affect circulating GLP-1 and PYY concentrations.^136^ This provides some evidence that acetate can have a direct effect on the central appetite network, independently of peripheral hormones.

#### Indole

Indole is mainly produced by colonic microorganisms through metabolism in animal bodies. Among the metabolites produced by the gut microbiota, indole can serve as a molecular signal that stimulates the secretion of GLP-1 in intestinal endocrine L cells, thereby regulating food intake and appetite^137,138^ (Figure 5). Recent studies have shown that indole derivatives, such as indole-3-ethanol (IEt), indole-3-pyruvic acid (IPyA), and indole-3-aldehyde (I3A), can also reduce intestinal permeability by binding to the aryl hydrocarbon receptor (AhR),^139^ which may contribute to intestinal barrier function and appetite control. Bansal et al. found that indole can upregulate the expression of genes related to mucosal barrier and mucin production, decrease NF-κB activity and the expression of the proinflammatory cytokine IL-8, while upregulating the expression of the anti-inflammatory cytokine IL-10.^140^ Recent research has demonstrated that indole can stimulate intestinal endocrine L cells to regulate the secretion of the gut incretin GLP-1. Indole increases GLP-1 secretion in the short term but suppresses it in the long term,^131^ suggesting that indole may regulate host appetite by influencing the secretion of gastrointestinal peptides.

##### Tryptophan

The gut microbiota plays a crucial role in controlling the availability and metabolism of tryptophan (Trp), indirectly or directly regulating metabolic balance and even affecting appetite.^141–144^ Tryptophan can both influence the secretion of intestinal hormones and cross the blood-brain barrier to directly activate satiety circuits in the brain.^145^ Numerous studies have explored the impact of dietary supplementation and reduced tryptophan intake on appetite control, but the results are inconsistent and even contradictory. For instance, animal studies have shown that tryptophan supplementation can stimulate food intake by enhancing gastrin, 5-hydroxytryptamine (5-HT), neuropeptide Y (NPY), and growth hormone-insulin-like growth factor (GH-IGF) signaling.^146,147^ However, other animal studies have demonstrated that a 5% tryptophan supplement in healthy rats can increase satiety and reduce feed intake.^148^ Additionally, severe tryptophan restriction can decrease plasma leptin and gastrin concentrations, increase plasma GLP-1 and PYY concentrations, leading to decreased food intake and weight loss, while moderate tryptophan restriction increases energy expenditure in obesity-prone rats.^149^

In a tryptophan restriction study by Arashdeep Singh et al., rats were fed normal chow, low-protein chow, 70% tryptophan, 40% tryptophan, or 10% tryptophan. The findings demonstrated a significant difference when compared to the control group, 40% and 10% tryptophan reduced food intake by 42% and 59%, respectively, throughout the restriction period. The 70% tryptophan group was comparable to the control. During the recovery period, the 40% treatment group transiently decreased food intake on day 27, while the 10% treatment group increased intake on day 24 but decreased it on days 23, 25, and 28. Severe tryptophan restriction reduced body weight, fat content, and lean body mass, while the 70% treatment group was comparable to the control. These findings suggest that the effect of tryptophan on appetite is complex and depends on both dietary tryptophan intake and the host’s metabolic state.

Overall, the regulation of tryptophan (Trp) metabolism by the gut microbiota is involved in the control of host appetite, although the effects are somewhat inconsistent, and the reasons are not yet clear. This complexity may be partially attributed to whether the stimulation of Trp reaches the “threshold” required to regulate appetite and energy intake. These discoveries encourage further exploration of the specific roles and mechanisms of Trp and its metabolites in the gut microbiota in appetite control.

#### Neurotransmitter

There is limited information about the relationships between neurotransmitters and microflora. Table 1 list some neurochemicals isolated from bacteria within the human gut.

**Table 1.** Representative list of neurochemicals isolated from bacteria within the human gut.^150^

Genus	Neurochemical
Lactobacillus, Bifidobacterium	GABA
Streptococcus, Escherichia, Enterococcus, Lactococcus, Lactobacillus	Serotonin
Escherichia, Bacillus	Norepinephrine
Escherichia, Bacillus, Lactococcus, Lactobacillus, Streptococcus	Dopamine
Escherichia, Bacillus, Lactococcus, Lactobacillus, Streptococcus	Acetylcholine
Lactococcus, Lactobacillus, Streptococcus, Enterococcus	Histamine

##### GABA

Gamma-aminobutyric acid (GABA) serves as a primary inhibitory neurotransmitter within the mammalian central nervous system (CNS). It can be produced in large amounts by gut microbiota such as Bacteroides, Parabacteroides, and Escherichia coli using dietary glutamate catalyzed by glutamic acid decarboxylase, and it has the function of regulating host appetite.^151^ GABA may promote host appetite through two mechanisms: (1) GABA binds to GABAA and GABAB receptors in the brain, thereby inhibiting the expression of anorexigenic neurons POMC;^152^ (2) GABA synergistically weakens melanocortin resistance with NPY and increases host food intake by binding to GABAA receptors in the paraventricular nucleus.^153^

Li, et al.^154^ found that gavage of 30 mg/kg of GABA significantly increased food intake in mice and upregulated the gene expression levels of GABA transaminase 2 and GABA-A receptor in the jejunum, while downregulating GLP-1 levels co-localized with GABA-A receptors. Simultaneously, it increased NPY activity and c-fos positive neuron expression in the ARC, NTS, and DVC, and decreased POMC activity. Subsequently, the researchers cut the vagus nerve of the mice and observed that compared to the sham-operated group, the food intake, NPY activity, and c-fos expression in the NTS and DVC of the vagus nerve-cut mice decreased significantly, while POMC activity increased.

These findings suggest that GABA produced by gut microbiota may regulate host appetite by interacting with specific receptors in the brain and altering the activity of neuropeptides such as NPY and POMC. Furthermore, the vagus nerve may play a crucial role in facilitating the modulation of GABA on appetite control. However, the exact mechanisms underlying these interactions and their physiological implications remain to be fully elucidated.

In addition, most studies examining the effects of GABA on host health have focused on dietary intake of GABA, rather than endogenous GABA production within the host.^155^ Protected GABA supplementation in ruminants has increased food intake in growing lambs and calves and suppressed CCK signaling,^156,157^ possibly due to the co-expression of GABA and CCK and the sharing of similar signaling pathways.^157–160^ Therefore, it seems reasonable to hypothesize that GABA may participate in appetite control by acting on its receptors in the gastrointestinal tract and brain, thereby affecting the secretion of intestinal hormones and the activation of central neurons, respectively. However, there is limited research on the relationship between GABA produced by gut microbiota and appetite control; further studies are needed to explore the role of GABA produced by gut microbiota in host metabolic health and determine whether GABA can cross the blood-brain barrier and regulate appetite in the central nervous system.

##### 5-HT

Approximately 90% of circulating 5-hydroxytryptamine (5-HT) is produced by endothelial cells (ECs) in the host intestine and then stored in circulating platelets, which transport 5-HT to every organ and tissue, including the brain.^161^ Although 5-HT cannot directly cross the blood-brain barrier, platelet-derived 5-HT can increase the level of 5-HT in the central nervous system, potentially linking intestinal 5-HT with brain function.^162^ Multiple studies have shown that 5-HT plays a crucial role in regulating energy metabolism and suppressing appetite through various mechanisms, including improving insulin sensitivity and regulating intestinal functions (such as motility, secretion, absorption, and sensation) by directly acting on the enteric nervous system and hypothalamic AgRP and POMC neurons. Therefore, the gut microbiota can significantly participate in appetite control by regulating intestinal and central 5-HT signaling. However, gut microbiota can also regulate serotonin turnover in the brain by altering the levels of serotonin precursors.^150,163^ Tryptophan is the most significant precursor for serotonin synthesis, and gut microbiota can synthesize tryptophan from scratch. Tryptophan in the peripheral system can cross the blood-brain barrier and participate in the synthesis of brain serotonin. Desbonnet et al. showed that Bifidobacterium can increase the concentration of tryptophan in the plasma of rats, affecting tryptophan metabolism.^164^ The hypothalamus is the primary central nervous system regulating host appetite, and various receptors expressed in the arcuate nucleus and paraventricular nucleus of the hypothalamus are believed to be involved in serotonin-regulated host feeding.

Later studies have shown that the binding of melanocortin to MC4R is necessary for 5-HT2CR to regulate appetite.^165^ After binding with 5-HT2CR, serotonin activates the expression of anorexigenic neurons POMC, producing α-MSH, which suppresses appetite.^166^ The latest research has shown that 5-HT1BR can inhibit the activity of orexigenic neurons NPY/AgRP, reducing appetite^167,168^ (Table 2).

**Table 2.** The relevant receptor of serotonin regulating appetite.

Receptor	Expression site	Deficiency	Reference
5-HT1BR	Arcuate nucleus (ARC)	Feed intake increased	^169^
5-HT2CR	Arcuate nucleus (ARC)	Feed intake increased	^169^
MC4R	Paraventricular nucleus(PVN)	Hyperphagia	^170^

#### Branched-chain amino acids (BCAAs)

Isoleucine (Ile), leucine (Leu), and valine (Val) are collectively referred to as BCAAs. These amino acids cannot be synthesized by the host itself and must be produced by gut microbiota such as Bacteroides, Streptococcus, and Lactobacillus species or obtained directly from food. They are essential amino acids (AA) involved in the regulation of food intake.^171^ Deficiency, excess, or imbalance in the ratio of BCAAs can lead to suppressed appetite, while a balanced ratio of BCAAs promotes host feeding. The regulation of food intake by BCAAs involves two pathways: on the one hand, BCAAs activate amino acid receptors, affecting the level of appetite factors; on the other hand, BCAAs regulate hypothalamic appetite pathways, thereby influencing neuronal expression.

Recent studies have found that adding Ile, Leu, and Val to low-protein diets to balance the BCAA levels in the diet can regulate the colonic microbiota structure of piglets, favoring the proliferation of beneficial bacteria such as Lactobacillus. Simultaneously, there is a significant increase in feed intake. Further validation has indicated that this effect is mainly mediated by the balance of Leu and Val. Zheng et al.^172^ also made similar discoveries and confirmed that the increase in feed intake of piglets is due to BCAAs acting as signaling molecules, activating the mammalian target of rapamycin cascade reaction in the hypothalamus and inhibiting the general AA control pathway, thereby upregulating the expression of NPY and AgRP neurons and downregulating the activity of melanocortin 4 receptor and CART.

Gloaguen et al. reported that a lack of Val in the diet can decrease feed intake in pigs, while the addition of extra Leu leads to an imbalance in the ratio of BCAAs, further exacerbating anorexia. Secondly, in an in vitro pig model study, pig jejunum tissue was stimulated with 0, 2.5, 5.0, 10.0, and 15.0 mmol/L of BCAAs. It was found that 10.0 mmol/L of BCAAs significantly increased the release of CCK and the mRNA and protein expression of taste receptor family 1 member 1/3 (T1R1/3). Furthermore, the researchers administered an inhibitor of T1R1/3 and observed a decrease in CCK and T1R1/3 levels. This indicates that BCAAs can induce the secretion of the satiety hormone CCK through the jejunum T1R1/3 and confirms that excessive supplementation of BCAAs can affect gastrointestinal feedback signals, thereby reducing host food intake.

#### Bile acids

Bile acids (BAs) are synthesized by the liver and released into the intestinal lumen during eating. They are transformed into secondary BAs, such as lithocholic acid (LCA) and deoxycholic acid (DCA), by the action of intestinal bacteria.^173^ Bile acid synthesis is influenced by gut microbiota, primarily depending on enzymes like CYP7A1 and CYP27A1.^174,175^ In addition to their roles in lipid metabolism and inflammation, secondary BAs act as signaling molecules, regulating the secretion of appetite hormones by activating TGR5 and FXR, thereby controlling appetite^176^ (Figure 5). Secondary BAs can activate TGR5 on intestinal L cells, influencing the secretion of other hormones.^177,178^ Furthermore, bile acids can directly bind to gastrointestinal receptors, regulating the secretion of appetite-related hormones. Alterations in bile acid composition, for instance, can stimulate the secretion of GLP-1 and PYY, leading to slower gastric emptying and decreased food intake.^179^

In summary, bile acid metabolism, influenced by gut microbiota, can regulate appetite-related hormones, thereby controlling appetite.^180,181^ Studies have shown that a BA mixture containing DCA can upregulate TGR5 expression, increase co-localized PYY and GLP-1, promote the binding of GLP-1 to pancreatic beta cell receptors, induce insulin production, and regulate feeding behavior.^179,179^ However, in TGR5 gene-knockout mice, no signal for the release of PYY and GLP-1 from EECs was detected after intestinal perfusion with BAs. Furthermore, the FXR signaling pathway is crucial for the modulation of appetite by bile acids (BAs).^180^ When intestinal FXR is activated, it increases the production of FGF15/19, which enters the systemic circulation, crosses the blood-brain barrier (BBB), binds to receptors in the brain, and exerts its effects.^180,181^ Injection of FGF19 into the lateral ventricles of mice leads to upregulation of FGFR activity in the ARC nucleus and decreased expression of NPY and AgRP genes.^181^ This suggests that the BA-FXR-FGF19 pathway can inhibit NPY/AgRP neuron activity, playing a crucial role in regulating the host’s eating behavior.

### Intestinal bacterial components

LPS, as the main component of the outer membrane of Gram-negative bacteria, is a potent endotoxin that can trigger inflammatory cascades upon entering the blood circulation. According to literature reports,^81^ a hyperlipidemic diet can stimulate the TLR4 inflammatory pathway by promoting the transport of LPS and the activation of lipopolysaccharide binding protein, thereby regulating the feeding center in the brain. A study on rats has shown^169^ that chronic low-dose LPS treatment can reduce the leptin signal transmitted by the vagus nerve, further decreasing the satiety induced by CCK. Gut microbiota plays a crucial role in regulating host appetite, and the release of LPS is considered one of the key factors promoting appetite^170^ (Figure 5). When the intestinal barrier function is compromised, a large amount of LPS crosses the intestinal barrier and enters the blood, leading to inflammation and possibly promoting appetite through the activation of relevant pathways, thereby contributing to obesity^170^ Moreover, the gut microbiota encompasses a diverse range of microorganisms, including bacteria, fungi, viruses, and archaea, can produce proteins with similar sequences to appetite-regulating peptides such as leptin, PYY, ghrelin, α-MSH, NPY, and AgRp. For example,^169^ the ClpB heat shock chaperone protein in Escherichia coli may exhibit a similar anorexic effect due to its sequence homology with the anorexia-related α-MSH (Figure 5). Research has shown that oral administration of Escherichia coli K12 to mice reduces food intake and body weight. However, this anorexic effect is significantly attenuated in ClpB-deficient Escherichia coli K12 mice, suggesting that ClpB may be involved in the reduction of food intake. Recent studies further indicate^170^ that food restriction increases plasma levels of ClpB, which is associated with increased relative abundance of Enterobacteriaceae bacteria and increased intestinal permeability, thereby enhancing satiety through the activation of anorexic neurons in mice.

It is worth noting that compared to lean individuals, the abundance of the ClpB gene from Enterobacteriaceae bacteria is lower in obese humans.^182^ This is consistent with previous findings that obese individuals have lower numbers of Enterobacteriaceae bacteria that synthesize ClpB. These results suggest that a decrease in the number of Enterobacteriaceae bacteria may be associated with obesity, and the production of ClpB may play a crucial role in this process. These research data support a possible mechanistic link between ClpB derived from gut microbiota and host appetite. However, it remains unclear whether other specific gut microbiota can produce ClpB and regulate appetite, warranting further exploration.

(**Path1**: gut microbes influence appetite hormone secretion : The appetite-regulating hormones transmit the fullness state from the intestine to the brain through hypothalamic NPY/Agrp and POMC neurons, thereby affecting the host’s eating behavior.

**Path2**: Intestinal microbial metabolites affect appetite: The gut microbiota produces a variety of metabolites, including SCFAs, secondary bile acids, branched-chain amino acids, etc. These metabolites can affect the host’s appetite by directly interacting with EEC cells.

**Path3**: Intestinal microbial components influence appetite: The gut microbiota can produce protein sequences identical to appetite-regulating peptides, such as ClpB, to regulate the secretion of anorexigenic hormones from intestinal endocrine cells.)

## Practice and application importance

### Animal production

From the perspective of animal production, the influence of gut microbiota on animal appetite holds significant practical relevance and application prospects. Appetite is a crucial factor that affects animal feed intake and production performance, and gut microbiota plays a pivotal role in regulating it. By modulating the structure and function of the gut microbiota, it is possible to influence animals’ feeding behavior and energy metabolism, thereby promoting animal health and enhancing production performance.

On one hand, gut microbiota can regulate animal appetite by influencing key pathways related to appetite. By identifying critical targets and signaling molecules involved in appetite regulation, it is possible to manipulate the interaction between the host’s appetite system and gut microbiota, controlling animals’ feed intake and selection, and enhancing feed utilization efficiency.

On the other hand, gut microbiota can also affect animal appetite through its interaction with the host’s immune system. The diversity and stability of the gut microbiota are crucial for the normal functioning of the host’s immune system. By modulating the gut microbiota, it is possible to enhance animals’ immunity, reduce the occurrence of diseases, and consequently improve their appetite and production performance.

In practical applications, methods such as the addition of probiotics and prebiotics can be used to regulate gut microbiota and improve animals’ appetite and production performance. For example, adding specific probiotics to feed can promote the growth of beneficial bacteria and inhibit the proliferation of harmful bacteria, thereby improving animals’ digestive function and appetite. Additionally, optimizing the structure and function of the gut microbiota through adjusted feed formulations and improved feeding management can further enhance animals’ appetite and production performance.

Looking ahead, with further research into the relationship between gut microbiota and animal appetite, we will be able to gain a more accurate understanding of the mechanism by which gut microbiota affects animal production. This will enable the development of more precise and effective regulatory strategies. These strategies will contribute to the sustainable development of animal production, improving animal welfare and production efficiency, and providing safer and healthier animal-derived food products for human consumption. Additionally, the integration of gut microbiota modulation into animal production systems offers the potential for reducing environmental impacts and enhancing the overall sustainability of the food production chain.

### Human diseases and health

From the perspective of human health, the impact of gut microbiota on appetite holds profound practical significance and application prospects. Appetite regulation is a crucial mechanism for maintaining energy balance and weight management, and gut microbiota plays a significant role in this process. By modulating the gut microbiota community, we can influence an individual’s appetite and energy intake, thereby promoting the maintenance of a healthy weight and the prevention of chronic diseases.

Gut microbiota interact with the host’s appetite regulatory system through the production of metabolites such as short-chain fatty acids (SCFA) and other bioactive molecules. These metabolites can influence the secretion of gastrointestinal hormones, such as glucagon-like peptide-1 (GLP-1) and ghrelin, thereby regulating appetite and satiety. For example, certain SCFAs can activate G-protein-coupled receptors in the gut, promoting the transmission of satiety signals and reducing food intake.

Moreover, the diversity and composition of gut microbiota are closely related to an individual’s appetite and weight management. Studies have shown that there are differences in the gut microbiota composition between obese individuals and lean individuals, with obese individuals exhibiting lower diversity and specific bacterial abundances. By modulating the structure and function of the gut microbiota community, it is possible to improve an individual’s appetite regulatory mechanism and promote the maintenance of a healthy weight.

In practical applications, the modulation of gut microbiota has become a new strategy for weight management and the prevention of chronic diseases. For instance, the intake of probiotics, prebiotics, or fecal microbiota transplantation can be used to regulate the gut microbiota community and improve an individual’s appetite and energy metabolism. These applications have broad prospects and hold the potential to provide new approaches and methods for the prevention and treatment of chronic diseases such as obesity and diabetes.

As research on the mechanism of gut microbiota and appetite regulation continues to deepen, we hope to gain a more accurate understanding of the role of gut microbiota in human health. This will lead to the development of more precise and personalized intervention strategies, which will have significant practical implications for human health. By promoting the maintenance of a healthy weight and the prevention of chronic diseases, we can improve people’s quality of life and overall health.

## Discussion

### Conclusion and prospects

There exists a tight interplay between the gut microbial flora and the nutrient-sensing receptors on the intestinal surface, which can be either direct or indirect. Through the communication of the gut-brain axis, these signals are transmitted to the central nervous system via the vagus nerve, peripheral circulatory system, or immune system, thereby influencing the host’s appetite. The formation of this “microbiota-gut-brain” axis reveals the crucial role of gut microbiota in appetite regulation. The regulatory signals of appetite primarily rely on appetite hormones and microbial metabolites.

However, despite our recognition of the influence of gut microbiota on the central nervous system of the host and its potential mechanisms in appetite regulation, further research is still needed. The diversity and complexity of gut microbiota and their metabolites make it difficult to precisely identify their regulatory targets and mechanisms. Currently, we cannot determine whether this regulation is driven by a single bacterium or the synergistic effect of multiple bacteria. Additionally, due to the individual differences in gut microbiota composition, as well as the influence of diet, environment, gene expression, and other factors on gut microbiota throughout the lifecycle, defining the optimal gut microbiota remains extremely challenging.

Therefore, further understanding of how specific members of gut microbiota participate in appetite control is crucial for developing new preventive and therapeutic interventions. As long as these microorganisms are beneficial to the host, they have the potential to constitute what we refer to as a healthy microbiota. Consequently, future research should focus on exploring the impact of gut microbiota changes and variations, aiming to provide us with a deeper understanding of the relationship between gut microbiota and host health.

In summary, the thorough research on the mechanism of gut microbiota and appetite regulation holds significant practical significance and application prospects. This not only helps to promote the sustainable development of animal production, improve animal welfare and production efficiency, but also allows us to develop more precise and personalized intervention strategies by gaining a deeper understanding of the role of gut microbiota in human health. These strategies aim to improve appetite regulation, promote the maintenance of a healthy weight, and prevent the occurrence of chronic diseases. These research achievements will make important contributions to the improvement of human health and quality of life.

## Abbreviations

NTS: Nucleus of the Solitary Tract
NPY: Neuropeptide Y
AgRP: Agouti-Related Protein
POMC: Proopiomelanocortin
CART: Cocaine and Amphetamine-Regulated Transcript Peptide
CNS: central nervous system
MDP: metabolite produced by gut microorganisms
SCFAs: short-chain fatty acids
GLP-1: glucagon-like peptide-1
PYY: peptide YY
BMI: body mass index
BBB: blood-brain barrier
ARC: arcuate nucleus
CCK: cholecystokinin
DPPIV: dipeptidyl peptidase IV
HFD: high-fat diet
BAs: bile acids
TLR4: toll-like receptor 4
IECs: intestinal epithelial cells
LPS: lipopolysaccharides
BCAAs: branched-chain amino acids
FFARs: free fatty acid receptors
ADFI: average daily feed intake
IEt: indole-3-ethanol
IPyA: indole-3-pyruvic acid
I3A: indole-3-aldehyde
AhR: hydrocarbon receptor
Trp: tryptophan
5-HT: 5-hydroxytryptamine
GH-IGF: growth hormone-insulin-like growth factor
GABA: Gamma-aminobutyric acid
PVN: Paraventricular nucleus
Ile: Isoleucine
Leu: leucine
Val: valine
AA: amino acids
DCA: deoxycholic acid

## Data Availability

The data that support the findings of this study are openly available in [repository name e.g “figshare”] at http://doi.org/10.1080/19490976.2024.2414796, reference number [reference number].

## Statement of significance

This review comprehensively summarizes relevant studies on the impact of gut microbiota on appetite in humans and animals, and collates the mechanisms through which gut microbiota affects appetite based on extensive research. The study of the influence of gut microbiota on appetite in humans and animals not only helps us to deeply understand the mysteries of life science, but also promises to provide new approaches and methods for improving the health status of humans and animals, as well as for the prevention and treatment of metabolic diseases.
